# Physicians’ awareness of oral-systemic links and its association with physician-reported patient referral to dentists

**DOI:** 10.1371/journal.pone.0276479

**Published:** 2022-10-20

**Authors:** Fatimah Al-Habib, Fatimah Al Abdulbaqi, Hala Alibrahim, Yasmin Alhamdan, Muhammad Ashraf Nazir, Jehan AlHumaid

**Affiliations:** 1 College of Dentistry, Imam Abdulrahman Bin Faisal University, Dammam, Saudi Arabia; 2 Department of Preventive Dental Science, College of Dentistry, Imam Abdulrahman Bin Faisal University, Dammam, Saudi Arabia; International Medical University, MALAYSIA

## Abstract

**Introduction:**

Physicians promote oral health by screening for oral pathologies, providing emergency help, and referring patients to dentists. The literature consistently reports a robust association between periodontal disease and systemic diseases. However, it is not fully known if physicians in Saudi Arabia are aware of the oral-systemic disease links. Therefore, the study aimed to evaluate physicians’ awareness of the oral-systemic disease links and their association with patients’ referral to dentists and patients’ access to oral care.

**Methods:**

This cross-sectional study was conducted (June 2019-January 2020) on physicians working in private and public hospitals in the Eastern province of Saudi Arabia. The physicians (n = 333) responded to a paper-based self-administered and pilot-tested questionnaire which inquired about their awareness of oral-systemic disease links and patient referral to dentists. The reliability of items of oral-systemic disease links was evaluated by calculating Cronbach’s alpha (0.84).

**Results:**

Most participants (92.8%) agreed/strongly agreed that oral health is associated with systemic health. Half of the sample (50.8%) referred patients to dentists during the last month. Almost 80.5% believed that more patients will be referred to the dentists because of their awareness of the oral-systemic disease links and 84% believed that more patients will access oral care services if patients were aware of the oral-systemic disease links. The participants who referred patients to the dentists during the last month (P = 0.038), believed that more patients will be referred (P = 0.001), and believed that more patients will access oral care (P = 0.003) demonstrated significantly higher oral-systemic disease links awareness score. The adjusted model showed patients’ referral to dentist (OR = 1.96.10, P = 0.006) and believing in more patients accessing oral care (OR = 2.05, P = 0.044) were associated with significantly increased awareness of the oral-systemic disease links.

**Conclusion:**

Most physicians in the study were aware of the oral-systemic disease links. Increased awareness of the oral-systemic disease links was significantly associated with the referral of patients to dentists and belief in improved patients’ access to oral care services.

## Introduction

Primary care physicians are the most important care providers in the healthcare system [1]. Over 90% of patients seek information about risk factors, diseases, and recommendations for treatment from their physicians [2]. They also promote oral health by screening for oral pathologies, providing emergency help, and referring patients to dentists. Many patients with dental pain also visit physicians and the emergency department for dental care [3]. Physicians can also play an important role in improving oral health by educating patients and providing preventive oral care through interdisciplinary approaches [3, 4].

The oral-systemic disease links refer to the connections between oral health and systemic health. The literature consistently reports the strong association between periodontal disease and systemic diseases including; diabetes, cardiovascular diseases, osteoporosis, respiratory diseases, and adverse pregnancy outcomes to name a few [5–9]. The oral cavity contains about 700 types of microorganisms which are related to diabetes, obesity, liver disease, and cancers [10]. These interconnections between oral and systemic diseases highlight the importance of treating these conditions through the collaborative efforts of dental and medical professionals [11].

The literature indicates the robust links between periodontal disease and atherosclerotic cardiovascular disease [8], diabetes [12], asthma, chronic obstructive pulmonary disease, pneumonia [7, 13], preeclampsia, low birth weight, and preterm birth [9, 14]. According to the International Diabetes Federation and the European Federation of Periodontology (2018), there is a bidirectional relationship between diabetes and periodontitis. Periodontal disease increases the risk of dysglycemia, insulin resistance, and incident type 2 diabetes, and periodontal treatment can reduce HbA1C in diabetic patients after 3 months [15]. A meta-analysis found a strong association between periodontal disease and increased risk of stroke [16]. Periodontal treatment is known to result in positive outcomes in patients with rheumatoid arthritis [17]. More than 100 systemic conditions and around 500 medications demonstrate oral manifestations [18]. Immune and inflammatory systems can play a role in the development of periodontal disease because many systemic factors have been shown to increase the destruction of periodontal tissues. For instance, if the number or function of polymorphonuclear leucocytes (PMNs) decrease, then the severity and rate of periodontal disease destruction increase [19].

However, many physicians fail to perform clinical examination of the oral cavity while attending patients in their clinics [20]. Moreover, physicians have inadequate knowledge and training about oral health [21, 22]. As a result, patients with oral problems are not referred by physicians to dental professionals and they don’t receive the proper dental care that they need. This leads to more oral problems, increased costs, and negative consequences for patients [1, 23]. There are also chances of fragmented care in case healthcare providers deliver the care only related to their specialty and therefore other health care issues can be overlooked [11]. Therefore, physicians should have adequate knowledge and skills to screen patients with oral problems and refer them to dental professionals. Developing an automated referral system can enable physicians refer more patients to receive dental care conveniently and improve physician-dentist communication and quality of patient care [1].

Similarly, dentists should also provide a few preventive medical services such as monitoring blood pressure and screening for diabetes [24]. For patients with smoking habits, dental professionals should raise awareness about tobacco use and tools for smoking cessation, and give patient referrals as needed. Regular screening of patients can also reveal early signs of certain systemic conditions and enhance patient referrals to physicians [11]. To provide optimal care to the patients, inter-professional care is needed as it provides a comprehensive treatment and so improves the patients’ overall health and reduce the healthcare cost [1].

Hein et al. assessed the inclusion of oral-systemic health in undergraduate programs in different English-speaking universities around the globe, including Australia, New Zealand, the United States, Canada, Europe, and Asia. The authors found that 59.6% of the respondents stated that inclusion of the oral-systemic health in their curricula was inadequate. Moreover, most of the students were not taught how to examine the oral cavity and were not instructed to perform a thorough oral examination [25].

There is a strong association between oral health and systemic health, and physicians can play an important role in oral health promotion. However, it is not fully known if physicians are aware of the latest evidence regarding the oral-systemic disease links. Similarly, there is a lack of data about the influence of their awareness of the oral-systemic disease links on patient referrals to dentists and patients’ access to oral care. Our study, therefore, evaluated physicians’ awareness of the oral-systemic disease links and their associations with patient referral to dentists.

### Alternative hypothesis

The physicians who have increased awareness of the oral-systemic disease links will refer more patients to dentists compared with those physicians who have low awareness.

## Methods

### Study design and participants

This cross-sectional study was conducted on physicians working in different cities of the Eastern province of Saudi Arabia. The data collection was completed in eight months (June 2019-January 2020). The physicians working in private and public hospitals provided their responses using a self-administered questionnaire. A sample of 351 participants was calculated based on margin of error (0.5), response distribution (50%), the population size of the physician (≈ 4,000), and significance level (0.95). http://www.raosoft.com/samplesize.html

A list of 22 hospitals located in Dammam, Dhahran, Khobar, Jubail, and AlQatif cities was obtained and hospitals were then selected randomly using the lottery method. The participants from selected hospitals were recruited using a convenience sampling technique. The study included general practitioners and specialists/consultants, whereas interns were excluded from the study. The physicians were approached in-person to have an adequate response rate of the study as a low response is obtained in online surveys. The self-administered questionnaires were delivered in their institutions, and if a physician was unable to provide response in the first visit due to his/her busy schedule, then he/she was approached after an interval of two-three weeks, and maximum of three visits were made.

### Study instrument

The questionnaire used in the study included questions divided into four sections. The first section was about personal information of physicians (age, gender, type of practice, qualification, graduation, number of years in clinical practice), oral health training in the undergraduate program, and continuing education course on oral health. There were 11 questions about oral-systemic disease links in the second section of the questionnaire. These questions were about the association between oral and systemic health, the association between periodontal disease and heart disease, stroke, adverse pregnancy outcomes, diabetes mellitus, rheumatoid arthritis, respiratory disease, kidney disease, and oral cancer, systemic diseases having oral manifestations, and the negative impact of oral disease on the quality of life. A five-point Likert scale (strongly disagree, disagree, neutral, agree, strongly agree) was used for these questions. The items related to the oral-systemic disease links were derived from a previous study where authors confirmed their validity and internal consistency (Cronbach alpha = 0.79) [26]. The third section included physician’s opinions about their willingness to educate patients, influence of awareness about oral-systemic disease links on patient referral to the dentists and patients’ access to oral care services, and barriers to patient education [17]. Most of these questions were answered by selecting Yes or No.

The questionnaire was reviewed by the research team and a senior public health faculty member from the college of medicine to confirm its relevance to physicians in Saudi Arabia. The reliability of the instrument (11 items related to the oral-systemic disease links) was calculated using Cronbach’s alpha (0.84) and was found satisfactory. Furthermore, the questionnaire was pretested for readability and ease of understanding survey items among 30 participants, and their responses were not used in the study.

### Ethical considerations

The study was approved from the ethical committee at Imam Abdulrahman Bin Faisal University. The approval of hospital administrators was secured before questionnaire administration in their respective institutions. The purpose of the study, its details, and potential benefits were discussed with study participants. The written informed and voluntary consent was obtained from them. They were assured about the confidentiality of their responses by using an anonymous questionnaire. The study followed the ethical principles for medical research according to the Helsinki Declaration.

### Statistical analysis

SPSS software (IBM SPSS Statistics for Windows, Version 22.0. Armonk, NY: IBM Corp) was used to perform statistical analysis. Descriptive statistics included means and standard deviations for continuous variables and frequencies and percentages for categorical variables. The scores of individual items in the questionnaire were summed to determine the score of the oral-systemic disease links which ranged from 5 to 55. The median value of the oral-systemic disease links score was used to dichotomize the variable into low (0–42) and high awareness (43–55) to evaluate its association with independent sociodemographic variables by calculating the odds ratio (OR) in bivariate analyses. Multiple logistic regression analysis was performed to calculate the adjusted odds ratio after controlling for other independent variables. The statistical significance level was set at <0.05.

## Results

The study included data of 333 participants with a mean age 41.88 (SD = 12.31) years. Most participants were males (59.8%), specialists/consultants (66.1%), worked in the public sector (76.3%), and had a clinical experience of more than 10 years (62.2%). Most of the participants believed in their important role in oral health promotion (94%) and were willing to educate their patients (88.6%). However, only 37.8% of the participants received oral health training in their undergraduate programs and 14.4% attended continuing education courses on oral health (Table 1).

**Table 1:** Demographic characteristics of physicians.

Variables	N = 333 N (%)
Gender:	
Male	199 (59.8)
Female	134 (40.2)
Type of practice:	
Private	79 (23.7)
Public	254 (76.3)
Qualification	
Physician	113 (33.9)
Specialist/consultant	220 (66.1)
Graduation from:	
Saudi Arabia	66 (19.8)
Abroad	267 (80.2)
Number of years in practice	
≤10 years	126 (37.8)
> 10 years	207 (62.2)
Received oral health training in the undergraduate program	126 (37.8)
Received continuing education course on oral health	48 (14.4)
Believed in the important role of physicians in oral health promotion	313 (94.0)
Willing to educate your patients about the oral-systemic disease links	295 (88.6)

Table 2 shows the distribution of physicians’ responses to the statements about the oral-systemic disease links. Most participants agreed/strongly agreed that oral health is associated with systemic health (92.8%), oral disease can negatively affect the quality of life (90.4%), and many systemic diseases have oral manifestations (90.1). On the other hand, the least agreed/strongly agreed responses included periodontal disease is associated with stroke (38.4%), chronic kidney disease (43.2%), and rheumatoid arthritis (43.5%).

**Table 2:** Distribution of physicians’ responses about the oral-systemic disease links.

Statements	Disagree/strongly disagree N (%)	Neutral N (%)	Agree/ strongly agree N (%)
Oral health is associated with systemic health.	14 (4.2)	10 (3.0)	309 (92.8)
Periodontal disease is associated with heart disease.	30 (9.0)	59 (17.7)	244 (73.3)
Periodontal disease is associated with stroke.	46 (13.8)	159 (47.7)	128 (38.4)
Periodontal disease is related to adverse pregnancy outcomes.	38 (11.4)	128 (38.4)	167 (50.2)
There is two-way relationship between periodontal disease & diabetes mellitus.	21 (6.3)	85 (25.5)	227 (68.2)
Periodontal disease is related to rheumatoid arthritis.	44 (13.2)	144 (43.2)	145 (43.5)
There is an association between periodontal disease and respiratory disease.	31 (9.3)	116 (34.8)	186 (55.9)
Periodontal disease is related to chronic kidney disease.	53 (15.9)	136 (40.8)	144 (43.2)
Links exist between periodontal disease and oral cancers.	14 (4.2)	74 (22.2)	245 (73.6)
Many systemic diseases have oral manifestations which further complicate oral and systemic diseases.	8 (2.4)	25 (7.5)	300 (90.1)
Oral disease can negatively affect quality of life of individuals.	15 (4.5)	17 (5.1)	301 (90.4)

Half of the sample (50.8%) referred patients to dentists during the last month. A vast majority of participants (80.5%) believed that more patients will be referred to the dentists if physicians were aware of the oral-systemic disease links. Eighty-four percent believed that more patients will access oral care services if patients are aware of the oral-systemic disease links. The mean oral-systemic disease links awareness score of the sample was 42.37 (SD = 6.47). The participants who referred patients to dentists during the last month had significantly higher mean oral-systemic disease links awareness score (43.09, SD 7.09) than those who did not refer (41.62, SD 5.68) (P = 0.038). The participants who believed that more patients will be referred to dentists because of physicians’ awareness of oral-systemic disease links (P = 0.001) and more patients will access oral care services if they are aware of oral-systemic disease links (P = 0.003) had significantly higher awareness than those who did not believe (Table 3). Further analysis showed that a significantly higher proportion of participants who believed that more patients will be referred to dentists because of physicians’ awareness of the oral-systemic disease links referred patients to dentist (54.1%) than those who did not believe (45.9%) (P = 0.013).

**Table 3:** Relationship of physicians’ awareness of the oral-systemic disease links with patient referral.

Variables	Frequency (%)	Mean Score (SD)	P-value
Referred patients to dentist during the last month			
Yes	169 (50.8)	43.09 (7.09)	0.038*
No	164 (49.2)	41.62 (5.68)	
Believed that more patients will be referred to the dentist because of physicians’ awareness of oral-systemic disease links			0.001*
Yes	268 (80.5)	42.97 (6.55)	
No	65(19.5)	39.89 (5.48)	
Believed that more patients will access oral care services if they are aware of oral-systemic disease links			0.003*
Yes	279 (84)	42.82 (6.41)	
No	54 (16)	40.0 (6.28	

* Statistically significant

**Fig 1:**
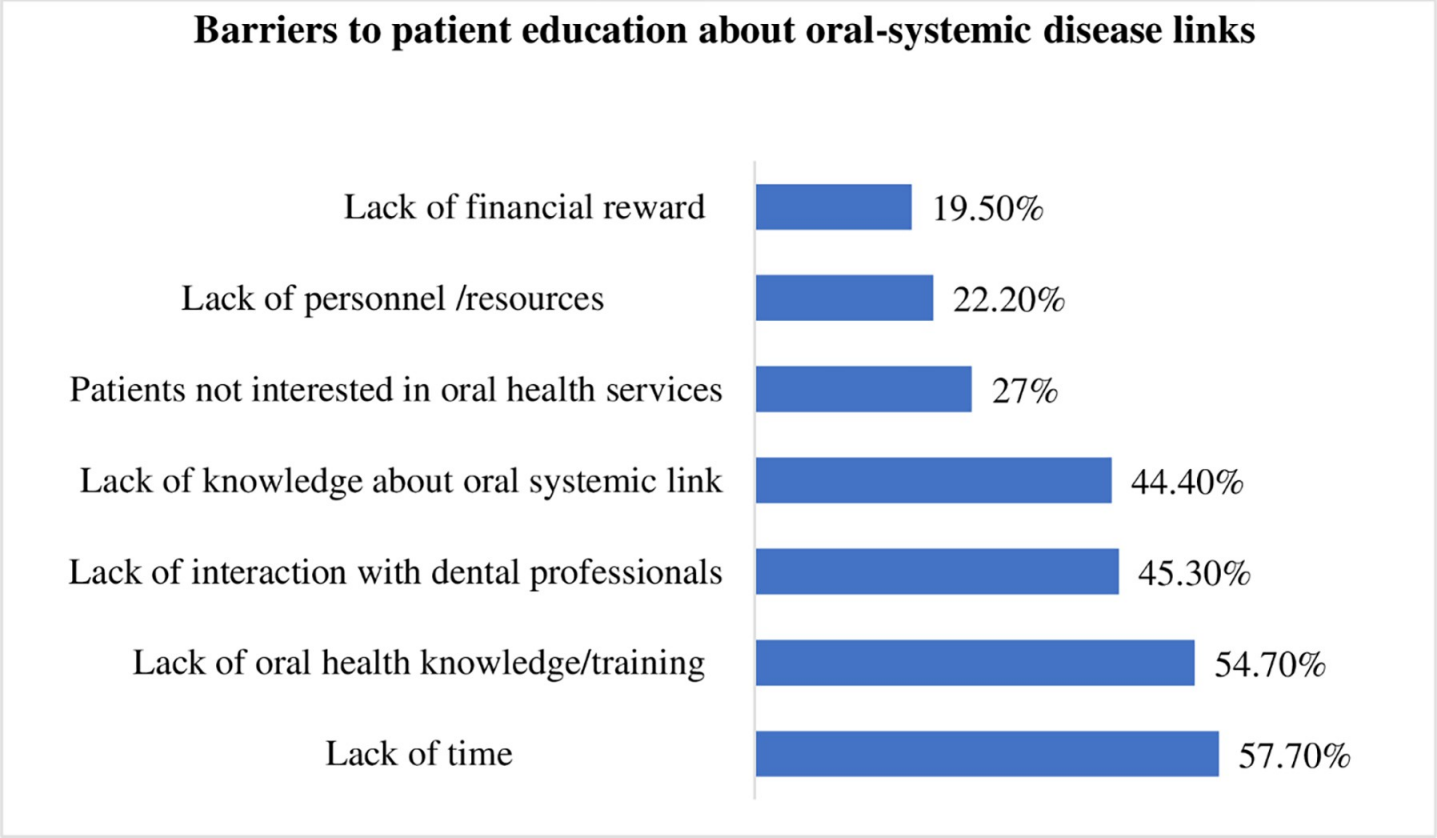
Barriers to providing education about the oral-systemic disease links to patients.

Analysis of barriers to patient education about the oral-systemic disease links is presented in Fig 1. The lack of time (57.7%), lack of oral health knowledge and training (54.7%), and lack of interactions with dental professionals (45.3%) were commonly reported barriers.

The sample consisted of 50.8% of participants with low and 49.2% with high awareness score of the oral-systemic disease links. The assumption of no multicolinearity for regression analysis was satisfied. Collinearity statistics showed that multicollinearity was not a problem in the regression analyses as no severe correlations between predictor variables were indicated by the variance inflation factor (VIF).

According to binary logistic regression analyses, recognizing the important role of physicians in oral health promotion (OR = 3.10, P = 0.025), referring patients to dentist during the last month (OR = 1.95.10, P = 0.003), believing that more patients will be referred to dentist (OR = 2.40.10, P = 0.002), and believing that more patients will access oral care services (OR = 2.68, P = 0.002) were significantly associated with increased awareness of the oral-systemic disease links.

Multiple logistic regression showed that the participants who referred patients to the dentist during the last month were significantly more likely (OR = 1.96.10, P = 0.006) to have increased awareness of the oral-systemic disease links than those who did not refer. Similarly, the participants who believed that more patients will access oral care services (OR = 2.05, P = 0.044) had significantly increased odds of awareness of the oral-systemic disease links than those who did not believe (Table 4).

**Table 4:** Binary and multiple logistic regression analyses: Factors associated with physicians’ awareness of the oral-systemic disease links.

	Binary logistic regression		Multiple logistic regression	
Variables	Unadjusted Odds Ratio	P-value	Adjusted Odds Ratio	P-value
Gender:	0.86 (0.55, 1.33)	0.502	0.83 (0.51, 1.37)	0.476
Male				
Female				
Type of practice:	0.94 (0.57, 1.56)	0.815	0.91 (0.41, 2.02)	0.808
Private				
Public				
Qualification	0.87 (0.55, 1.37)	0.539	0.85 (0.48, 1.49)	0.571
Physician				
Specialist/consultant				
Graduation from:	1.21 (0.71, 2.07)	0.493	2.29 (0.97, 5.45)	0.060
Saudi Arabia				
Abroad				
Number of years in practice	0.70 (0.45, 1.09)	0.111	0.61 (0.35, 1.08)	0.092
≤10 years				
> 10 years				
Received oral health training in undergraduate program	1.05 (0.67, 1.63)	0.831	1.08 (0.63, 1.85)	0.772
Received continuing education course on oral health	0.70 (0.38, 1.30)	0.256	0.72 (0.35, 1.51)	0.384
Recognized the important role of physicians in oral health promotion	3.10 (1.10, 8.73)	0.025*	1.92 (0.60, 6.18)	0.272
Willing to educate your patients about oral-systemic disease links	0.97 (0.49, 1.90)	0.922	0.65 (0.30, 1.42)	0.280
Referred patients to the dentist during the last month	1.95 (1.26, 3.02)	0.003*	1.96 (1.21, 3.17)	0.006*
Believed that more patients will be referred to the dentist because of physicians’ awareness of the oral-systemic disease links	2.40 (1.35, 4.25)	0.002*	1.75 (0.90, 3.38)	0.097
Believed that more patients will access oral care services if they are aware of the oral-systemic disease links	2.68 (1.43, 5.04)	0.002*	2.05 (1.02, 4.11)	0.044*

* Statistically significant

## Discussion

The association between oral health and systemic health was recognized by 92.8% of physicians in the present study. The study showed an association between increased awareness of the oral-systemic disease links among physicians and their referral of patients to dentists. The results also clearly suggested that physicians who referred patients to dentists were almost twice more likely to have significantly higher awareness of the oral-systemic disease links than those who did not refer patients. The association between physicians’ awareness of the oral-systemic disease links and patients’ referral is understandable because a strong association exists between periodontal disease and systemic diseases [5–9].

The present study also showed that a vast majority of physicians believed that more patients would access oral care if patients were aware of the oral-systemic disease links. Similar results were obtained in a previous study where 97% of dentists believed in greater utilization of oral care with patients’ awareness of the oral-systemic disease links [26]. Improved awareness of the oral-systemic disease links among physicians may lead to patients’ education for optimal oral health and patients may visit the dentist and utilize oral care services more frequently if they know the connection between oral health and systemic health.

In the study, 50% of physicians referred patients to the dentist during the last month. According to a study by Miloro et al, 74% of physicians referred patients to the dentist in the previous year [27]. On the other hand, 11.9% of physicians referred patients to the dentist and most agreed that delayed referral of dental treatment can result in life threating condition [23]. It was found that most physicians only give their patients the name of a dentist without following up with their referral and only half communicated with dentists to make an appointment for referred patients [19].

In the present study, more than half of physicians did not receive oral health training in their undergraduate programs. Previous studies have cited physicians’ lack of training in oral health care in the U.S [27], Saudi Arabia [21], and Iran [22]. As a result of inadequate oral health training of physicians, patients visiting emergency department or physicians receive prescriptions of antibiotics and painkillers rather getting comprehensive dental care, and such repeated visits may lead to antibiotic resistance and opiate dependency for patients with dental pain [3].

Similarly, most participants in this study did not receive continuing education courses on oral health, although the majority recognized their important role in oral health promotion. The present study also found a lack of oral health knowledge and training and a lack of interactions with dental professionals as barriers to patient education. These study findings call for providing opportunities to improve oral health knowledge through updating curricula of undergraduate medical programs, organizing continuing professional activities, and establishing inter-professional engagements between medical and dental professionals.

The findings of the present study may be used to improve physicians’ awareness of the oral-systemic disease links to enhance patient referral to dentists for positive oral health outcomes. However, the study has some limitations which should be considered while interpreting study results. This cross-sectional study observed significant associations between independent and dependent variables, however, this study design cannot be used to establish cause-effect relationships. Therefore, the impact of awareness of the oral-systemic disease links on patient referral to dentists and patients’ utilization of oral care should be further investigated in large longitudinal studies. The use of convenience sample and data collection from physicians in a province compromise its generalizability to physicians in other parts of Saudi Arabia. Additionally, there is the possibility of over-reporting of some responses due to social desirability bias. For instance, physicians may provide exaggerated responses about patients’ referral in the presence of researchers from dentistry. The items on oral-systemic disease links broadly focused on the association of periodontal disease with systemic disease. Although literature supports the association of dental caries with systemic disease particularly metabolic disorders [28]. The study used “median split” to categorize oral-systemic disease links awareness score into low and high awareness categories. However, the use of median split is often criticized because this may add error in the interpretation of results. For example, the participants who have a score just above the median cutoff are treated the same as those who are close to the maximum value on the scale. Therefore, results should be interpreted with caution.

## Conclusion

The study found that most physicians were aware of the oral-systemic disease links. A vast majority of physicians believed that more patients will be referred to the dentists because of their awareness of oral-systemic disease links. They also believed that more patients will access oral care services if patients are aware of the oral-systemic disease links. There was a significant relationship between the awareness of the oral-systemic disease links and patients’ referral to dentists. The physicians’ referral of patients to dentists and their belief in more patients’ accessing oral care were significantly associated with increased awareness of the oral-systemic disease links. Public health programs for oral health improvement should focus on providing opportunities to educate physicians about oral-systemic disease links and raise its awareness among their patients so that patients are referred to dentists for comprehensive oral care. These programs should raise awareness particularly about the association between periodontal disease and diabetes, heart disease, oral cancers, and respiratory disease. Physicians should also be educated about oral manifestations of systemic diseases and the impact of oral diseases on the quality of life.

## Supporting information

S1 Data (SAV)
